# Supplementary material for: Recessive mutations in muscle-specific isoforms of FXR1 cause congenital multi-minicore myopathy
Source: Nat Commun. 2019 Feb 15;10:797. doi: 10.1038/s41467-019-08548-9 (PMC6377633; doi:10.1038/s41467-019-08548-9)
Supplement: Supplementary file 1 — Supplementary Data 1 [file 41467_2019_8548_MOESM1_ESM.pdf]

SUPPLEMENTARY DATA

RECESSIVE MUTATIONS IN MUSCLE-SPECIFIC ISOFORMS OF FXR1 CAUSE CONGENITAL MULTI-MINICORE MYOPATHY

Maria Cristina Estañ, Elisa Fernández-Núñez, et al.

Supplementary Data

Detailed clinical description of patients

Family 1

The proband of family 1 was a 2.5 months old Egyptian male patient born to healthy first cousin parents. Family history included a similarly affected female sibling who died at the age of 70 days and a previous miscarriage. This sibling was delivered by Caesarean section, as oligohydramnios and reduced foetal movement were observed during pregnancy. Respiratory distress was noticed after birth and the baby was admitted to the neonatal intensive care unit for 21 days. Recurrent fractures of the humerus and femur, short neck, displaced low set nipples, severe hypotonia, hyperlaxity of joints, and ulnar deviation of hands were in the patient records. Laboratory investigations revealed normal calcium, phosphorus, alkaline phosphatase, and liver and renal function tests. Karyotype was 46, XX. Abdominal ultrasound was normal and echocardiography identified a small atrial septal defect. Skeletal surveys showed fractures of the long bones with normal callus formation.

The proband was also delivered at full term by Caesarean section. During pregnancy the mother was on anticoagulant therapy to increase foetal blood flow. Reduced foetal movement was noted and serial ultrasounds revealed diminished amniotic fluid at 32 weeks of gestational (Amniotic fluid index = 7cm) indicating oligohydramnios, but there were no detectable anomalies and the fetus had normal growth and morphology. After delivery, the patient was admitted to neonatal intensive care unit for a month due to severe hypotonia and multiple fractures of long bones with episodes of unexplained tachycardia that required propranolol ($0.5 mg \cdot kg^{-1}$) every 8 hours. On examination, the patient was very alert with good eye contact and followed objects appropriately. He was severely hypotonic, had a very weak cry, shallow breathing and was fed by Ryle tube because of weak oromotor dysfunction and severe choking spells. On examination head circumference was 39 cm (50th centile), weight 4.9 kg (50th centile) and length 55cm (-0.5 s.d.). Generally, the baby was not dysmorphic, but he had a short neck and skeletal deformities. There was severe joint

laxity with no obvious demarcation of large joints (hips, knees and elbows) except for a minor crease. The hands were opened with normal creases, ulnar deviated and hyperextended fingers. The feet were deviated laterally and dorsiflexed with fanning of the toes. There were bowed femurs due to malunion after fracture. The genitalia were hypoplastic with nearly absent scrotal sac. Abdominal ultrasound identified the testes very high in the inguinal canal. Skeletal survey showed fractures in humeri and femora with normal callus formation. The baby died at the age of five months.

Family 2

Sibling II-3

Sibling II-3, the eldest member of this sibship, is a 28 year old male with longstanding proximal muscle weakness. He was conceived naturally to healthy parents and the pregnancy was normal, with no polyhydramnios or decreased fetal movements, maternal diabetes, pregnancy-induced hypertension, trauma or infection during pregnancy. He was born at 36 weeks' gestation via spontaneous vaginal delivery and birth weight was 2.6 kg. He was noted to have bilateral cryptorchidism and hypotonia at birth. He had two episodes of lower respiratory tract infection and apnea in the first month of life, but otherwise was well. Developmental history was significant for gross motor delay; he sat at 1 year 9 months and walked at 2 years, 3 months. He has had difficulty climbing stairs and getting up and out of bed from a very young age. He has never had difficulty with fine motor skills or language. He has never had any learning difficulties and his hearing and vision have always been normal. He has never had episodes of encephalopathy, seizures, loss of consciousness, myoglobinuria or cramps, or any fluctuation of weakness. He had dyspnea on exertion but no palpitations or other cardiac symptoms, and serial echocardiograms have always been normal.

He underwent surgery at 18 months for bilateral orchidopexy, but otherwise has had no other admissions to hospital, and he takes no medications. Currently at age 28 years he continues to

have proximal muscle weakness and difficulties performing motor tasks such as getting up from a chair, squatting position, transferring from a standing position to the couch and climbing stairs. However, he is able to perform all activities of daily living but is slow, especially in dressing. He has no swallowing difficulties and his speech is normal. Sleep studies revealed moderate obstructive sleep apnea, for which he is treated with nocturnal BiPAP. He is currently enrolled in a post-secondary program at a community college.

On examination his height was 156 cm, weight was 87 kg and his head circumference was 57 cm. His cranial nerve examination was normal; specifically, there was no facial weakness and the extraocular movements were full. He had prominent calves and the tendo-achilles tendon was tight. Muscle tone was generally hypotonic and formal manual muscle power testing was as follows: neck flexors 4-, neck extensors 4, bilaterally, deltoids and biceps 3+ bilaterally, infraspinatus 4- bilaterally, supinator 4+ bilaterally, triceps 4 bilaterally, wrist flexors 5 bilaterally, wrist extensors, digit flexors, digit extensors and opponens pollicis 4+ bilaterally, interossei 4+ bilaterally. Iliopsoas, thigh adductors, thigh abductors were 2 bilaterally, as was gluteus maximus. Quadriceps was 4 bilaterally, hamstrings 4+ bilaterally, tibialis anterior 4+ bilaterally, gastrocnemius and soleus 5 bilaterally, extensor hallucis longus 4+ bilaterally and tibialis posterior and peronei 5 bilaterally. Cerebellar examination was normal and there was no evidence of incoordination or ataxia. His deep tendon reflexes were 1+ in biceps, triceps, knee and ankle bilaterally. His plantar response was flexor bilaterally. He had normal sensation to light touch, pain, vibration and proprioception. There was mild thoracolumbar scoliosis, with convexity towards the right side but no spinal rigidity. He had a positive Gowers sign and also walked with a Trendelenburg gait.

<u>Sibling II-4</u>

Sibling II-4, the middle member of this sibship, is a 26 year old male with proximal muscle weakness. He was born following a normal pregnancy at term via Caesarean section for prolonged labour. He was noted to be hypotonic at birth, but otherwise had a normal neonatal

period and there were no feeding or respiratory difficulties. His developmental history is significant for gross motor delay. He sat at 8 – 9 months of age, crawled after 12 months, and walked after 18 months of age. He was able to walk upstairs by 3 years and down stairs around 4-5 years. He cannot run or jump. Fine motor skills, language and speech milestones were achieved at the appropriate times. He has never had any episodes of encephalopathy, seizures, abnormal hearing, or abnormal vision. He has never had any learning difficulties or disability, and completed education at the college level. Currently at age 26 years his main difficulty is performing motor tasks which require rising from a sitting position or climbing stairs. He can walk for 90 minutes or longer with intermittent rest. He is independent for activities of daily living including cutting food, handling utensils, dressing and personal hygiene, but is slow. He has no swallowing difficulties or choking, and bladder and bowel function is normal. He does not have any history of palpitations, chest pain or difficulty breathing, and cardiac evaluations have always been normal. Sleep studies revealed mild obstructive sleep apnea but he has never required BiPAP treatment. He has had no hospitalizations or surgeries. He has recently been diagnosed with depression treated with citalopram 40 mg daily. He is currently employed as a chemical lab technician.

On examination, his height was 177 cm, weight was 90 kg and head circumference was 59 cm. Cranial nerves II to XII were normal and there was no evidence of ophthalmoplegia or ptosis. He had normal saccades, smooth pursuit, convergence and visual fields bilaterally. Muscle tone was globally hypotonic and the calves felt firm.

On examination of muscle strength, he required considerable support to transition from a sitting position from a supine position. Formal manual muscle testing was as follows: neck flexors 4-, neck extensors 4+, deltoids 3+ bilaterally, biceps 4 bilaterally, supinator 4 bilaterally, infraspinous 4– bilaterally, triceps 4– bilaterally, wrist flexors 4+ bilaterally, wrist extensors 4+ bilaterally, digit flexors 4+ bilaterally, digit extensors 4+ bilaterally, opponens pollicis 4+, flexor digitorum indicis and abductor digit minimi 4+ bilaterally. Iliopsoas and thigh abductors were 1 bilaterally; thigh adductors 2 bilaterally, gluteus maximus 3 bilaterally, quadriceps 4 bilaterally, hamstrings were 4+

bilaterally, tibialis anterior 4 bilaterally, gastrocnemius 4+ bilaterally, extensor hallucis longus 4 bilaterally and tibialis posterior and peronei 5 bilaterally. Cerebellar examination revealed normal coordination and he did not have any ataxia. His deep tendon reflexes were 1+ bilaterally. His plantar responses were flexor bilaterally. There were no sensory abnormalities to light touch, pain, vibration and proprioception. He had a positive Gowers sign and had a Trendelenburg gait. He had no scoliosis or scapular winging.

Sibling II-5

Sibling II-5, the youngest member of this sibship, is a 24 year old female. She was born following a normal pregnancy via elective Cesarean section. She was noted to be hypotonic at birth but otherwise had no feeding, cardiac or respiratory issues.

Her development is characterized by gross motor delays; she sat at 8 months, walked at 22 months, and was able to climb stairs at 4-5 years of age. Currently at age 24 years, she continues to have difficulty getting up and out of bed, climbing upstairs, or rise from the floor or from chairs. She is unable to walk quickly, run, jump, or skip. She cannot ride a bicycle but she can exercise on a stationary bicycle. With regard to exercise endurance, she can walk for thirty minutes without feeling fatigued. She does not have cramps, myoglobinuria, or muscle stiffness but does feel some pain in her muscles the day following intense physical activity. She is independent with respect to all activities of daily living. She has never had fluctuating or fatigable weakness, episodes of myoglobinuria, bladder or bowel difficulties, chest pain, palpitations or difficulty breathing. Her sleep studies have revealed mild obstructive sleep apnea but she has never required BiPAP treatment. She has been diagnosed with bipolar disorder, social anxiety disorder, generalized anxiety disorder, attention deficit disorder, and has a past history of bulimia, and is under the care of a psychiatrist. Current medications include aripiprazole 10 mg daily, lisdexamfetamine 60 mg daily, and fluoxetine 20mg daily.

On physical examination her height was 155 cm, weight 66 kilograms and head circumference 55.7 cm. There was no evidence of facial dysmorphism and had a normal skin and subcutaneous

tissue. Examination of cranial nerves X to XII was normal. She did not have nystagmus or ptosis. Extraocular movements were full in all cardinal gaze directions. Saccades, smooth pursuit, convergence and visual fields were normal. Her fundi did not reveal any evidence of disc edema. Her calf muscles were firm but there were no palpable muscle fibrosis or muscle hypertrophy. Muscle tone was hypotonic. Formal manual muscle strength testing was as follows: neck flexors 4-, neck extensors 4+, deltoid 3+ bilaterally, biceps 4- bilaterally, supinators 4+ bilaterally, triceps 4- bilaterally, wrist flexors, wrist extensors, digit flexors and digit extensors 4+ bilaterally, opponens pollicis 5 bilaterally, abductor digiti minimi and flexor digitorum indicis 4 bilaterally. Iliopsoas was 3 bilaterally, thigh adductors 4 bilaterally, thigh abductors 4- bilaterally, gluteus maximus 3 bilaterally, quadriceps 4 bilaterally, hamstrings 4+ bilaterally, tibialis anterior 4+ bilaterally, gastrocnemius-soleus 4+ bilaterally, extensor hallucis longus 4+ bilaterally tibialis posterior and peronei 5 bilaterally. Cerebellar examination was normal. Deep tendon reflexes were 1+ bilaterally and plantar responses flexor. Sensory examination to light touch, pain, vibration and proprioception was normal. She had a positive Gowers sign and walked with a mild Trendelenburg gait. There was no evidence of any scoliosis or contractures of the paraspinal muscles.

Family History

All three siblings were conceived naturally to healthy non-consanguineous parents. The mother had two spontaneous pregnancy losses before the birth of this sibship. The maternal branch of the family is of Argentinean descent, and there are four maternal uncles and three maternal aunts who are healthy. One maternal female cousin has recently been diagnosed with muscular disease, however further clinical details are unavailable. The father is deceased and the paternal branch of the family is of Turkish descent. There is no significant history of congenital anomalies, other individuals with neuromuscular disorders or other inherited conditions in the family.

Biochemical and genetic tests in family 2

CK levels, thyroid studies and basic metabolic tests were normal. Microarray analysis and targeted molecular testing of *RYR1*, *SEPN1*, *TTN* and *ACTA1* were negative.
