# Supplementary material for: Recessive mutations in muscle-specific isoforms of FXR1 cause congenital multi-minicore myopathy
Source: Nat Commun. 2019 Feb 15;10:797. doi: 10.1038/s41467-019-08548-9 (PMC6377633; doi:10.1038/s41467-019-08548-9)
Supplement: Supplementary file 2 — Supplementary Information [file 41467_2019_8548_MOESM2_ESM.pdf]

SUPPLEMENTARY INFORMATION

RECESSIVE MUTATIONS IN MUSCLE-SPECIFIC ISOFORMS OF FXR1 CAUSE CONGENITAL MULTI-MINICORE MYOPATHY

María Cristina Estañ, Elisa Fernández-Núñez et al.

Supplementary Figures

Supplementary Figure 1

a

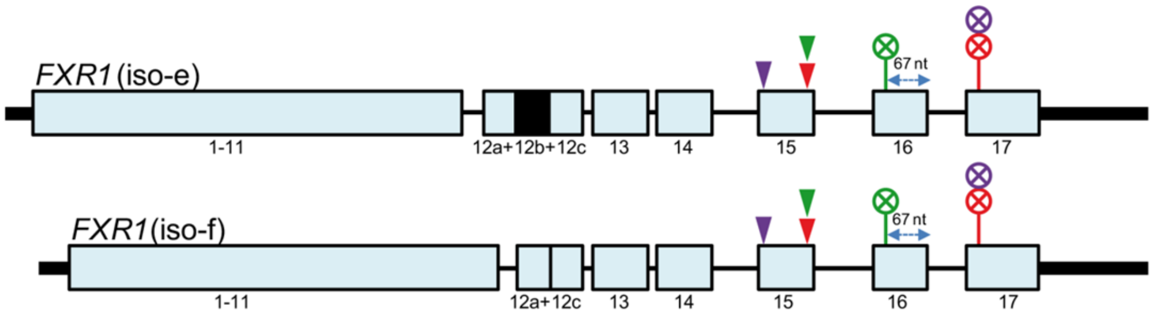

b

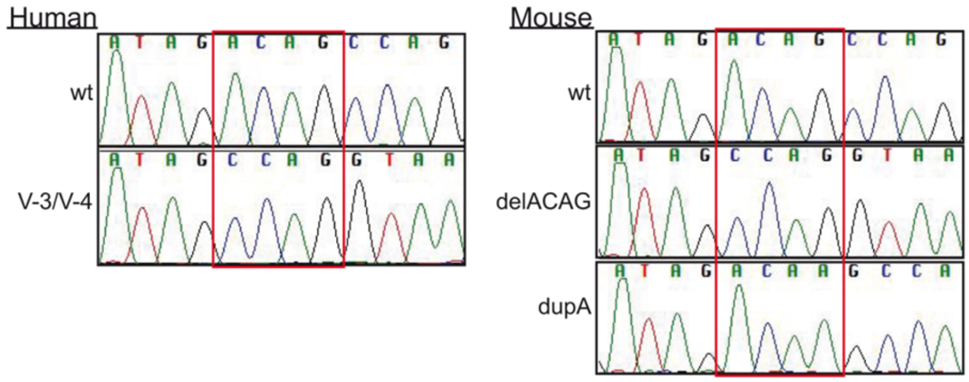

c

FXR1 isoforms (iso)		accesion number	mutation	protein effect	number of residues	position of stop codon	predicted MW (KDa)
human iso-e WT		XM_005247813.3	-	-	677	exon 17	76.19
human iso-f WT		XM_005247815.3	-	-	648	exon 17	73.12
family 1	human iso-e delACAG	XM_005247813.3	c.1764_1767delACAG	p.Arg588Serfs*37	623	exon 17	70.60
family 1	human iso-f delACAG	XM_005247815.3	c.1677_1680delACAG	p.Arg559Serfs*37	594	exon 17	67.58
family 2	human iso-e delA	XM_005247813.3	c.1707delA	p.Lys569Asnfs*57	624	exon 17	70.15
family 2	human iso-f delA	XM_005247815.3	c.1620delA	p.Lys540Asnfs*57	595	exon 17	67.08
mouse iso-e WT		NM_001113188.1	-	-	677	exon 17	76.22
mouse iso-f WT		XM_006535401.1	-	-	648	exon 17	73.12
mouse iso-e delACAG		NM_001113188.1	c.1764_1767delACAG	p.Arg588Serfs*37	623	exon 17	70.71
mouse iso-f delACAG		XM_006535401.1	c.1677_1680delACAG	p.Arg559Serfs*37	594	exon 17	67.61
mouse iso-e dupA		NM_001113188.1	c.1766dupA	p.Pro590Alafs*10	598	exon 16	67.73
mouse iso-f dupA		XM_006535401.1	c.1679dupA	p.Pro561Alafs*10	569	exon 16	64.63

d

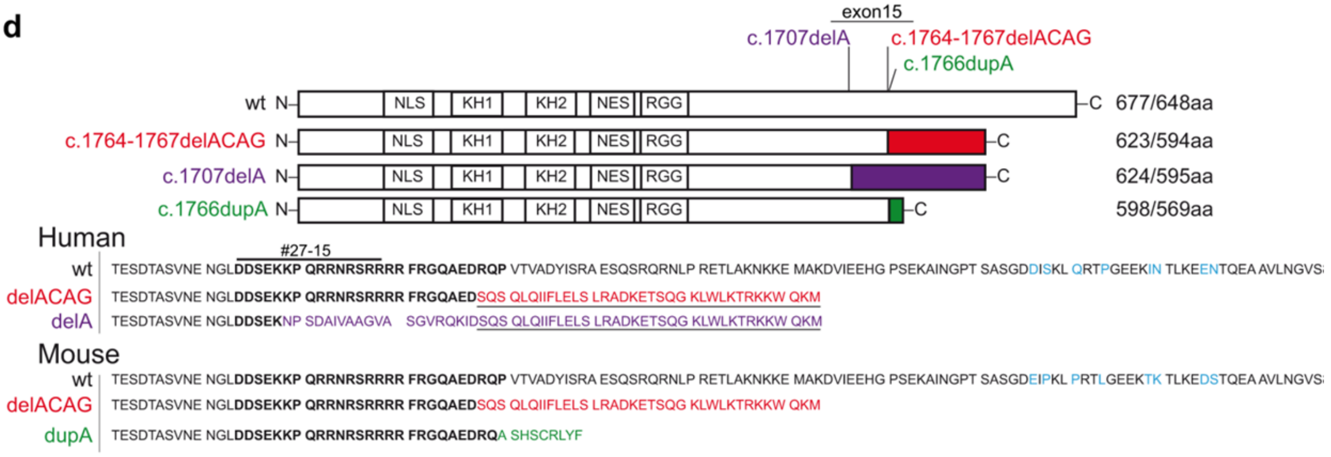

Supplementary Figure 1. Mutation description in patients and mice. a) Schematic representation of exons 12-17 (independent boxes) of *FXR1* iso-e and iso-f encoding P82,84. The central part of exon-12 (black box: 12b) is spliced out in iso-f. Exon structure of *FXR1* P70-80 isoforms is in Dube et al[1] and Kirpatrick et al[2]. Exon-15 frameshift variants are indicated by purple (delA), red (delACAG) and green (dupA) arrowheads and corresponding premature termination codons (PTC) generated by these mutations are shown with encircled crosses in identical colour. DelA and delACAG share the same PTC at the 5´-end of the terminal exon-17. DupA PTC is 67 nucleotides (nt) upstream of the last exon-exon junction and thus it elicits NMD[3]. b) DNA-sequencing chromatograms from a normal control (wt) and patients of family 1 (V-3 and V-4) containing the homozygous ACAG nucleotide deletion (squared in red) are shown next to equivalent exon-15 mouse sequence from wt, delACAG and dupA homozygotes. The single adenine duplicated in dupA mice is comprised within the four nucleotides deleted in delACAG mutants. c) Description of mutations from patients and mice showing iso-e/f reference sequences, mutation position, protein effect, total number of residues, position of PTCs and molecular weight of predicted mutant proteins. d) Upper panel: schematic representation of normal (wt) and mutant proteins showing localization of mutations, functional domains as described by Dube et al[1] and effect on the protein of each mutation. The delACAG (family 1 and delACAG mice), delA (family2) and dupA (dupA mice) variants cause the replacement of the last 90, 109 and 88 C-terminal amino acids of normal P82,84 by 36 (red box), 56 (purple box) and 9 (green box) new residues respectively. Bottom panel: amino acid sequence alignment of the C-terminal end of wt and mutant iso-e/f from humans and mice. New residues incorporated due to delACAG, delA or dupA mutations are in red, purple and green respectively. Blue letters designate non-identical amino acids between human and mouse. Exon-15 amino acids are in bold. DelACAG and delA proteins share their last 37 residues (underlined amino acids). The peptide used to raise the #27-15 antibody is indicated[1].

Supplementary Figure 2

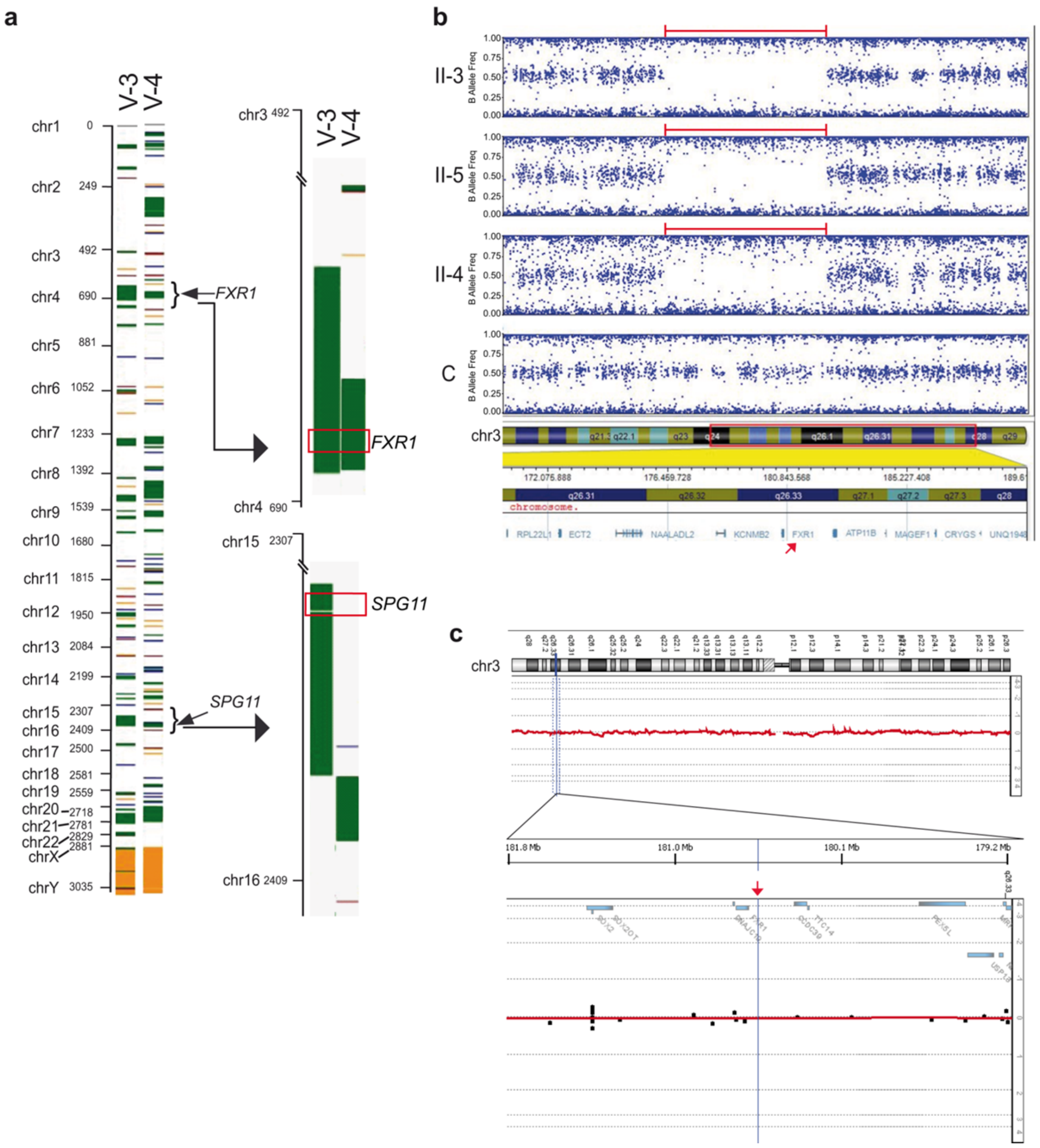

Supplementary Figure 2. SNP-arrays and aCGH in families 1 and 2. a) SNP-array hybridization of the proband (V-3) and affected fetus (V-4) of family 1 showing *FXR1* within large regions of homozygosity (ROH; green boxes) in V-3 (54.6 Mb) and V-4 (23.7 Mb) on chromosome 3. *SPG11* (chromosome 15, Supplementary Table 1b) is not within a ROH in V-4. b) B-allele frequency obtained by whole genome SNP-array hybridization in the three affected siblings of family 2 (II-3-4-5) and a control individual (C) revealing a common region of homozygosity of 5.98 Mb on chromosome 3 (red lines) comprising *FXR1* (red arrow) in the tree patients. B-allele plots correspond to a fragment of chromosome 3 spanning from q26.31 to q28. Images in a-b were obtained with GenomeStudio software (Illumina). c) aCGH (60,000 probes) for chromosome 3 corresponding to one of the patients of family 2 demonstrating no heterozygous deletion in the region encompassing *FXR1*. Images were obtained with Workbench Standard Edition 5.0 software (Agilent Technologies).

Supplementary Figure 3

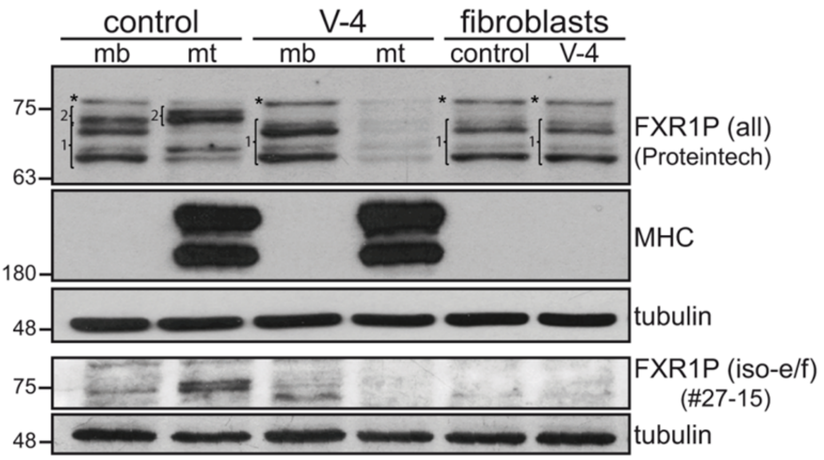

Supplementary Figure 3. FXR1P expression in V-4 primary cells. Representative immunoblot showing expression of FXR1P isoforms in the soluble fraction (SF) of protein extracts from control and V-4 myoblasts (mb), myotubes (mt) and fibroblasts. Numbered brackets indicate P70-80 (1) and P82,84 (2) isoforms. Note absence of P82,84 in the SF of V-4 cells. No variations in the levels of FXR1P isoforms were detected between control and V-4 fibroblasts. An antibody detecting all FXR1P isoforms (Proteintech) was used in the upper panel and another one specific for FXR1P isoforms e/f (#27-15) was used in the lower panel. MHC and tubulin served as controls for myotube differentiation and protein loading respectively. Asterisks denote a non-specific band. n=3.

Supplementary Figure 4

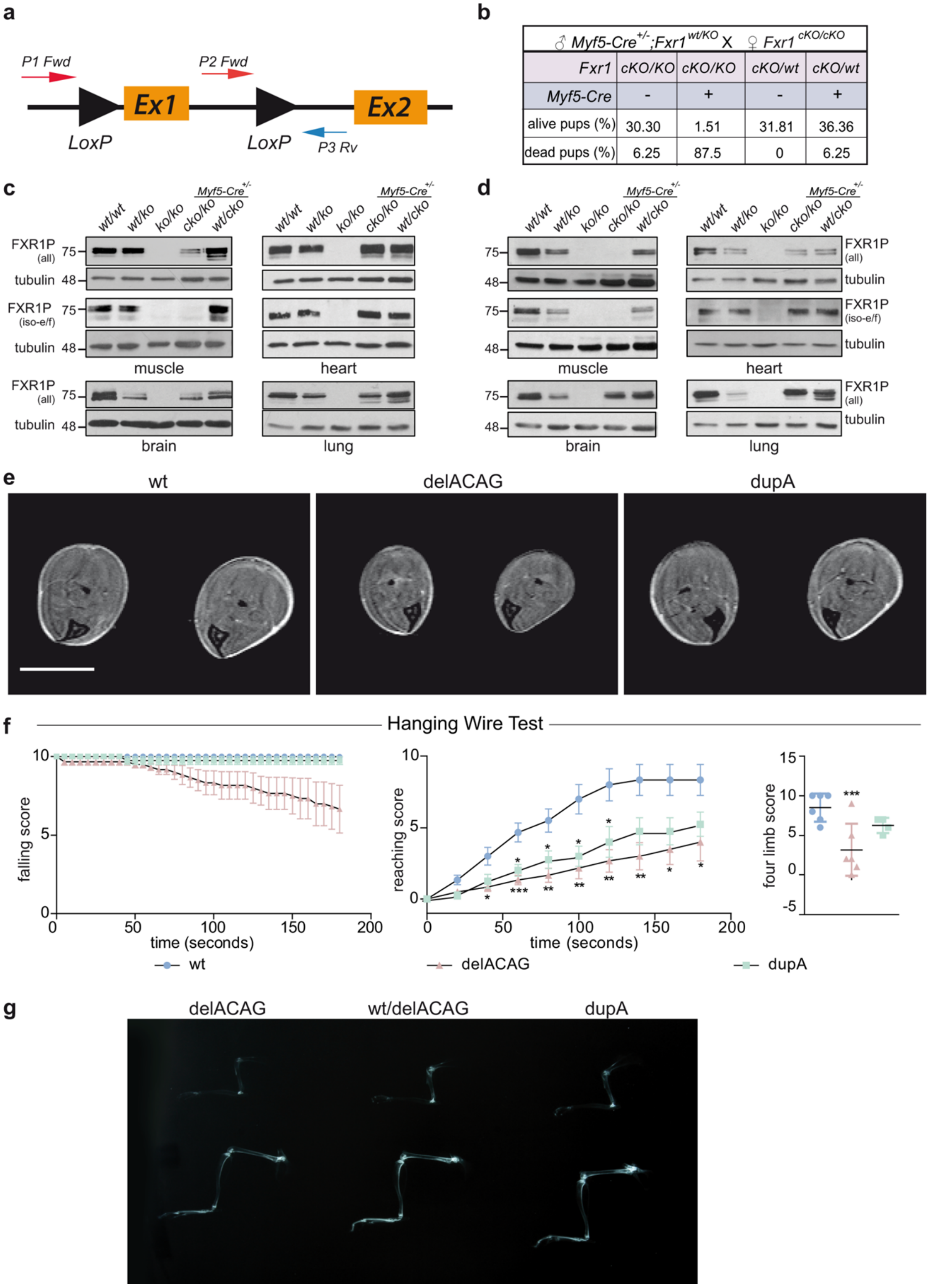

♂ Myf5-Cre+/-;Fxr1 wt/KO X ♀ Fxr1 cKO/cKO				
Fxr1	cKO/KO	cKO/KO	cKO/wt	cKO/wt
Myf5-Cre	-	+	-	+
alive pups (%)	30.30	1.51	31.81	36.36
dead pups (%)	6.25	87.5	0	6.25

Supplementary Figure 4. Phenotypes of *Fxr1*-conditional knockouts and exon-15 mutant mice. a) Schematic representation of *Fxr1* floxed allele. Exon-1 is flanked by two *LoxP* sites and the primers used for genotyping (P1-P3 and P2-P3) are indicated. P1-P3 yielded a PCR product only after Cre-mediated excision of exon-1 (ko allele), while positive product amplification using P2-P3 occurred in wt and non Cre-recombined alleles (cko). PCR products from wt and cko alleles differed in size due to *LoxP*. b) Relative frequency of each genotype respecting the total number of surviving pups or the total number of dead pups at P0-1 (n=82) from crosses between $Fxr1^{cko/cko}$ females and $Myf5\text{-}Cre^{+/-};Fxr1^{wt/ko}$ males. Presence or absence of *Myf5-Cre* is indicated with + and – symbols. c) Representative immunoblots using an anti-FXR1P antibody against all FXR1P isoforms (#ML13 antibody); or specific for isoforms e and f (#27-17 antibody) in skeletal muscle, heart, brain and lung protein extracts from E18.5 *Fxr1* conditional embryos (cko/ko or wt/cko) heterozygous for *Myf5-Cre* ($Myf5\text{-}Cre^{+/-}$). Tissue extracts from wild type (wt/wt), homozygous (ko/ko) and heterozygous (wt/ko) E18.5 embryos for a constitutive *Fxr1* null allele (exon-1 deleted) were included as controls. d) Same as c but using protein extracts from independent embryos. n=4. e) Representative image of a specific hindlimb MRI slice (three slices below the tibial plateau) from wt, delACAG and dupA mice illustrating differences in muscle mass. n= 4(wt), 9(delACAG), 4(dupA) mice. Scale bar 5 mm. f) From left to right: falling, reaching and four limb scoring graphs corresponding to the hanging wire test showing deficient performance in delACAG and intermediate results in dupA mice. Note that although the falling score was not statistical significant, delACAG mutants had lower values than wt and dupA mice. n= 6(wt), 6(delACAG), 4(dupA) mice. Graphs indicate mean ± s.e.m.(falling and reaching test) or mean ± s.d. (four limb score) followed by one-way ANOVA with Tukey post-hoc test. g) X-rays of left fore- and hind-limbs from delACAG, wt/delACAG and dupA mice taken simultaneously. n= 4(wt), 1(wt/delACAG), 8(delACAG), 4 (dupA) mice.

Supplementary Figure 5

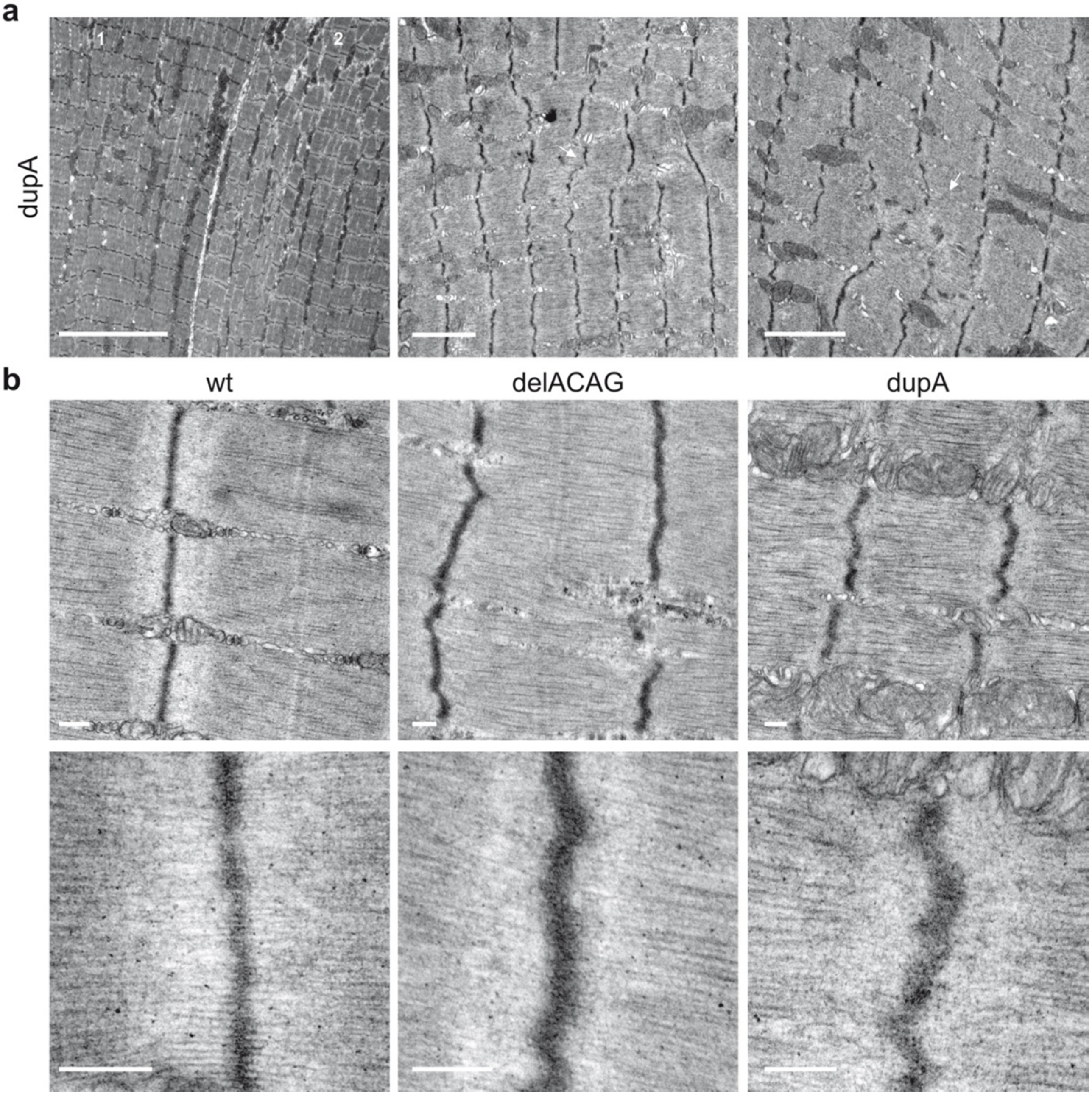

Supplementary Figure 5. TEM analysis of myofibres from exon-15 mutant mice. a) Representative TEM images of fibres from gastrocnemius of dupA mice. Numbers indicate different fibres and arrows point to areas of Z-streaming. Scale bars: 10 μm left panel, 2 μm middle and right panel. b) High magnification TEM images of gastrocnemius fibres demonstrating zigzagging, shortening and broadening of Z-bands in delACAG and dupA mutants compared to wt mice. Scale bars 200 nm. n= 3(wt), 4(delACAG), 2(dupA) mice.

Supplementary Figure 6

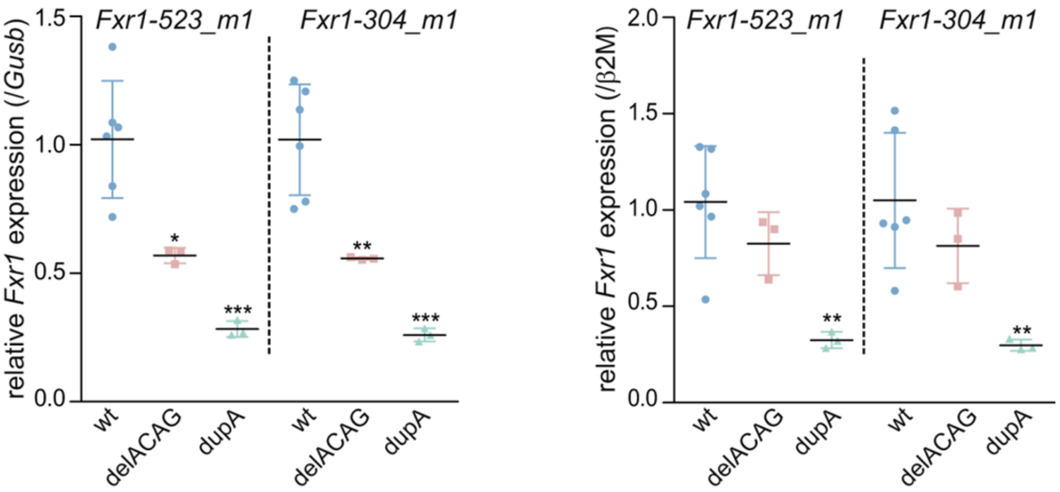

Supplementary Figure 6. qRT-PCR analysis of P82,84 mRNA levels in exon-15 mutant mice. Relative *Fxr1* expression using two different TaqMan expression assays in gastrocnemius of wt, delACAG and dupA mice at age 2.5 months. *Fxr1-523_m1* (exons 2-3; Mm00484523_m1) and *Fxr1-304_m1* (exons 14-15; Mm01286304_m1).Values are normalized to *Gusb* (left graph) or *β2M* (right graph) mRNA levels and expressed as fold change of the mean value of wt mice. n= 6(wt), 3(delACAG) and 3(dupA) mice. Graphs are mean ± s.d. followed by one-way ANOVA with Tukey post-hoc test.

Supplementary Figure 7

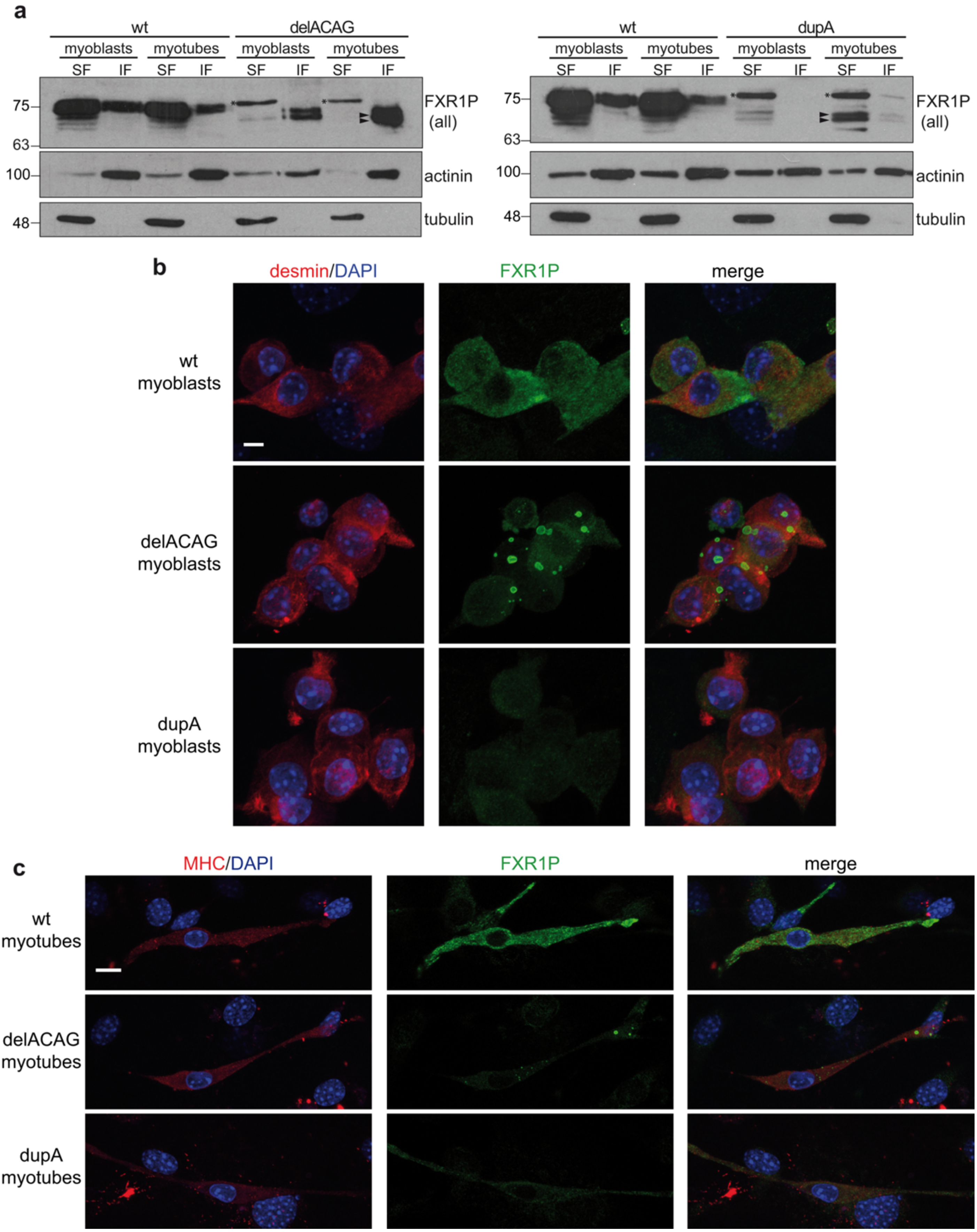

Supplementary Figure 7. FXR1P expression in primary myogenic cells from mutant mice. a) Representative WB of soluble (SF) and insoluble (IF) fractions from primary proliferating myoblasts and differentiated myotubes derived from wt, delACAG (left panel) and dupA (right panel) mice. Mutant P82,84 variants are indicated by arrowheads. DupA P82,84 variants were in the SF of cell extracts although, as in muscle tissue, required a long time of exposure to be detected. DelACAG P82,84 isoforms were in the IF of cell lysates. A number of 3 (left panel) and 2 (right panel) technical replicates of this experiment were performed using cells derived from 3(wt), 3(delACAG) and 1(dupA) mice. Asterisks denote a non-specific band. b-c) Confocal immunofluorescence analysis (Z-Project) of FXR1P in cultured myoblasts (b) and myotubes (c) from wt, delACAG and dupA mice showing ring-shaped FXR1P granules exclusively in delACAG cells since the myoblast stage. Note the reduction in FXR1P staining in dupA cells consistent with low protein levels of their corresponding P82,84 variants. Desmin and MHC expression was used as myogenic markers, with MHC being an indicator of myotube differentiation. Nuclei were labelled with DAPI. Primary myoblast cultures from n= 4(wt), 4(delACAG), 2(dupA) mice were analysed. Scale bars 5 µm in b, 10 µm in c.

Supplementary Figure 8

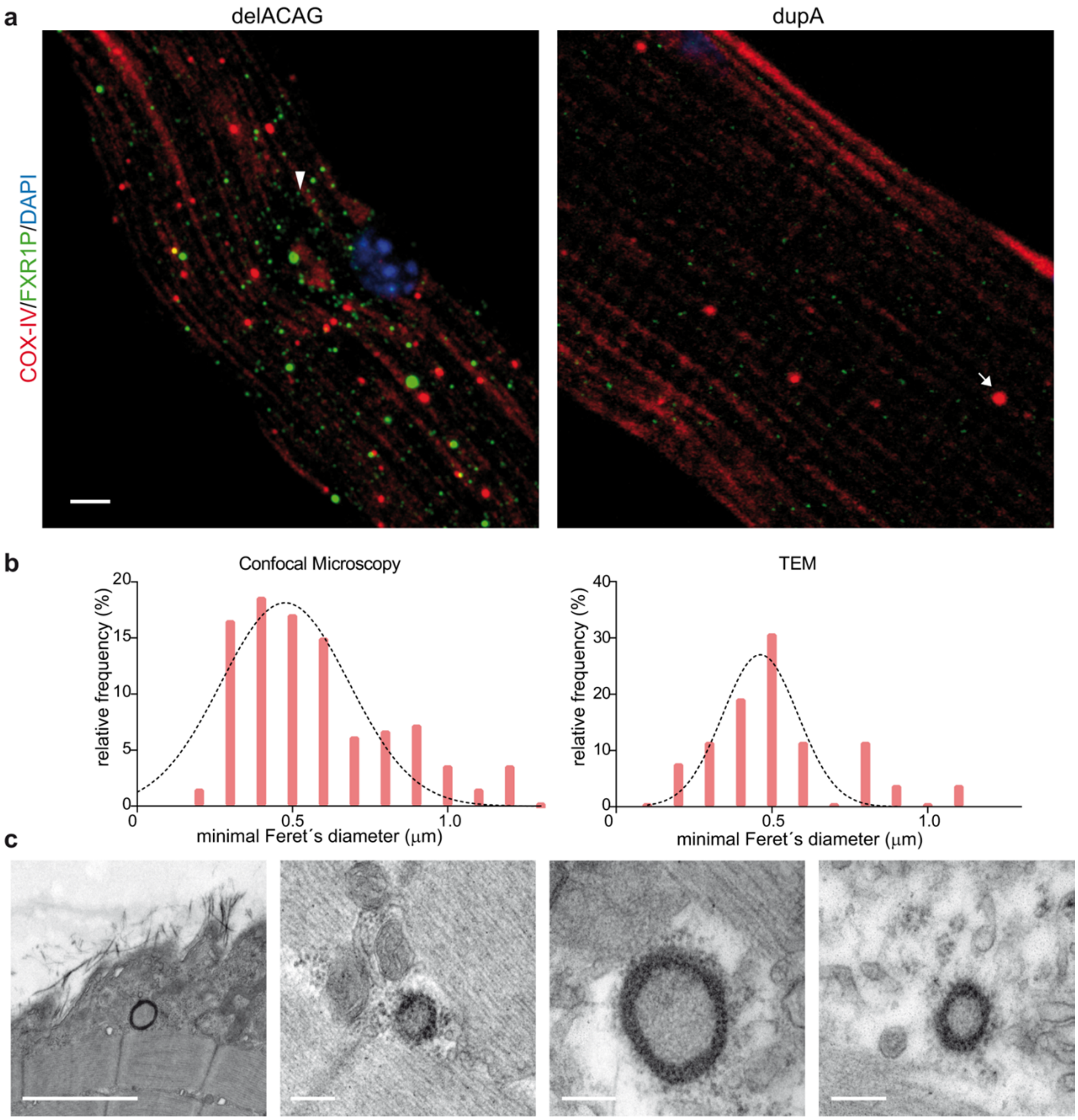

Supplementary Figure 8. Subcellular localization studies of P82,84 variants in exon-15 mutant mice. a) Maximum Z-project confocal co-immunofluorescence images of FXR1P (green) and COX-IV (red) in isolated EDL fibres from delACAG (left) and dupA (right) mice. Scale bar 5 µm. The arrowhead in delACAG denotes a core lesion lacking COX-IV staining. The arrow in dupA indicates abnormal mitochondrial accumulation. Nuclei are stained with DAPI (blue). b) Quantitative size determination of granules detected by anti-FXR1P confocal microscopy (CF) (left histogram) and TEM (right histogram) in fibres from delACAG mice. The relative frequency of FXR1P granules within a specific length range (bin-range: 0.1 µm) of minimal Feret's diameter (X-axis) is represented in the Y-axis. Total number of granules analysed: n=193 (CF) and n=26 (TEM). c) Representative TEM images corresponding to ultrathin sections of gastrocnemius from heterozygous wt/delACAG mice showing normal sarcomere structure and ring-shaped cytoplasmic granules. Scale bars: 2 µm left panel, 200 nm rest of panels.

Supplementary Figure 9

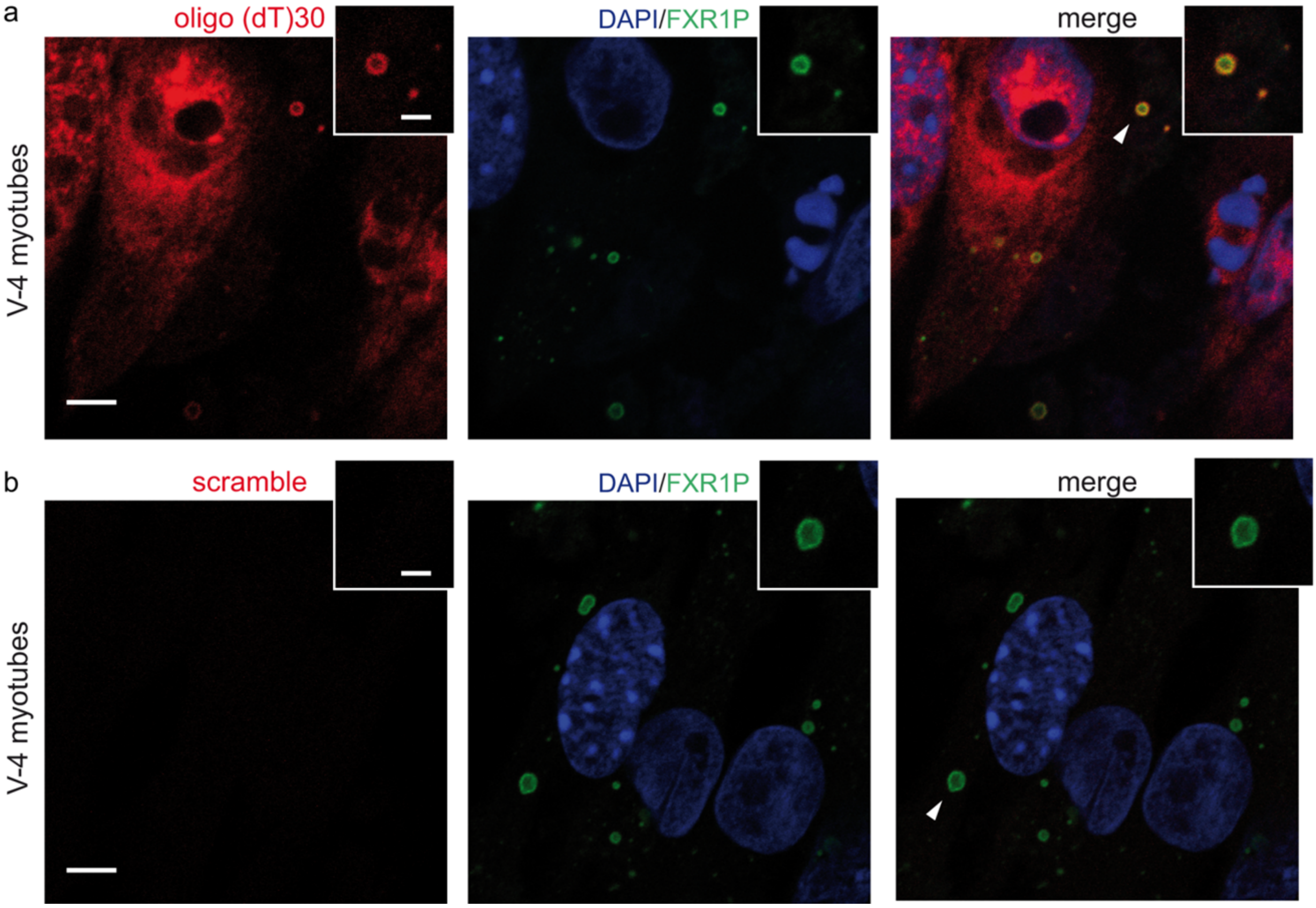

Supplementary Figure 9. V-4 delACAG granules incorporate mRNA. a-b) Confocal images (single Z-slice) of RNA-FISH performed in V-4 myotubes using Cy3-oligo(dT) (a) or a Cy3-scramble control probe (b) followed by anti-FXR1P immunostaining proving positive oligo (dT) signal in delACAG granules. Scale bars 5 µm. Arrowheads mark the areas shown with higher magnification. Scale bar 2µm. Nuclei were stained with DAPI. Three technical replicates of this experiment were performed.

Supplementary Figure 10

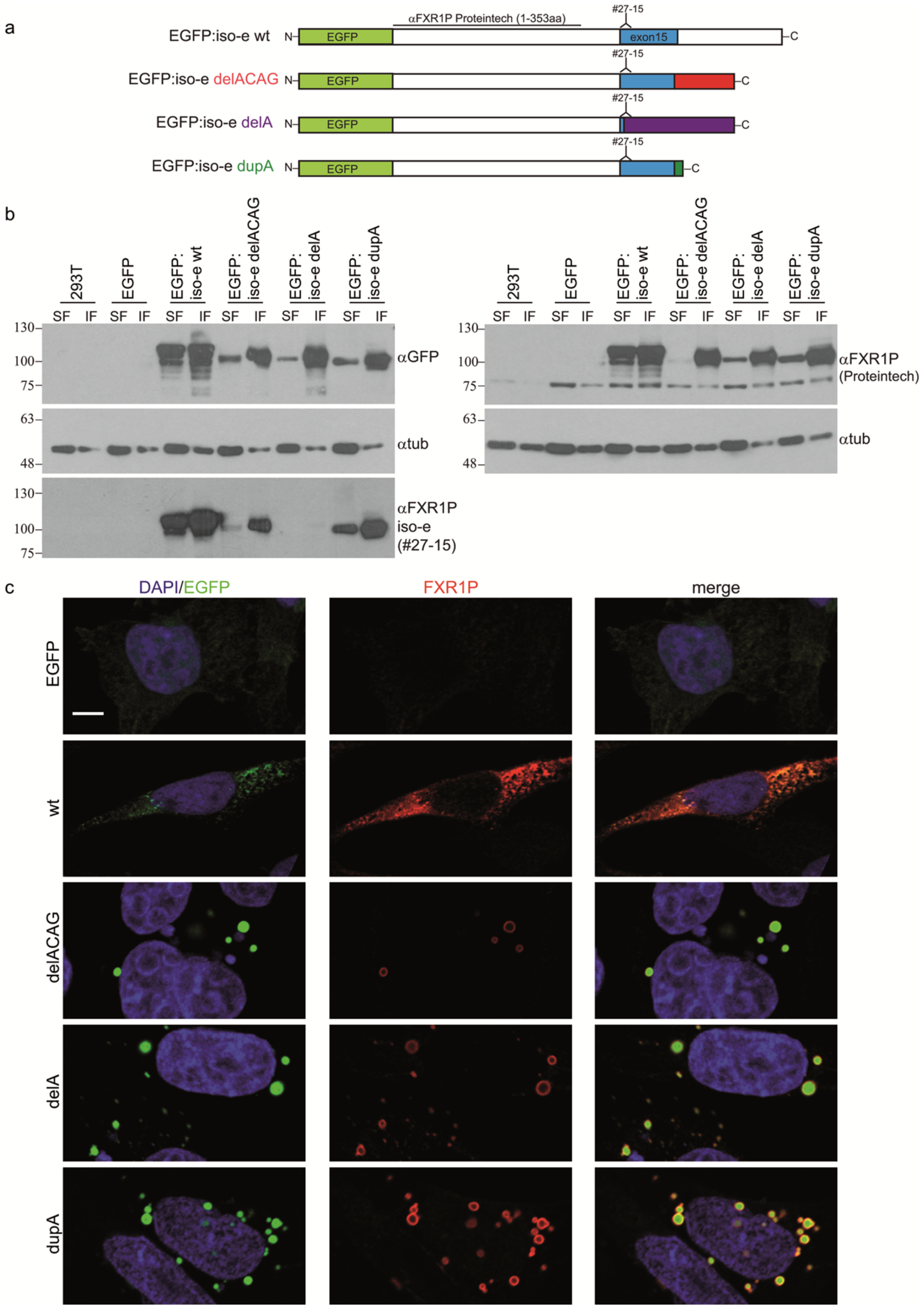

Supplementary Figure 10. Mutant iso-e isoforms assemble in ring-shaped granules in HeLa cells. a) Schematic representation of wild type and mutant EGFP:FXR1P-iso-e (human) fusion proteins showing exon-15 (blue box) and different C-terminal ends of the mutant proteins according to their respective mutations. Epitope position for Proteintech and #27-15 anti-FXR1P antibodies is indicated. b) Representative immunoblots from HEK293T protein extracts (soluble (SF) and insoluble (IF) fractions) demonstrating detection of the fusion proteins only in cells transfected with the EGFP:iso-e constructs indicated in a, but not in cells transfected with the empty pEGFP-C1 vector (EGFP), or in non-transfected cells (293T). Equally sized bands were identified by anti-FXR1P (Proteintech) raised against the N-terminal region of FXR1P (right panel) and by anti-GFP (left panel). The anti-GFP blot was stripped and reprobed with the exon-15 specific antibody #27-15 yielding an identical result except for EGFP:iso-e-delA (botton left panel). The delA variant only includes the first 5 amino acids of the peptide used to generate #27-15[1], and thus the corresponding fusion protein is not recognized by this antibody. In contrast, the full #27-15 immunogenic peptide is present in delACAG and dupA isoforms (Supplementary Fig. 1). Tubulin (αtub) was used as loading control. n=2. c) Dual confocal image (single Z-slice) for EGFP and anti-FXR1P staining (Proteintech) corresponding to HeLa cells transfected with the EGFP empty vector (EGFP) or with the different EGFP:iso-e constructs indicated in panel a showing ring-shaped FXR1P granules in cells transfected with all three mutant variants. n=3. Scale bar 5 μm. Nuclei are labelled with DAPI.

Supplementary Figure 11

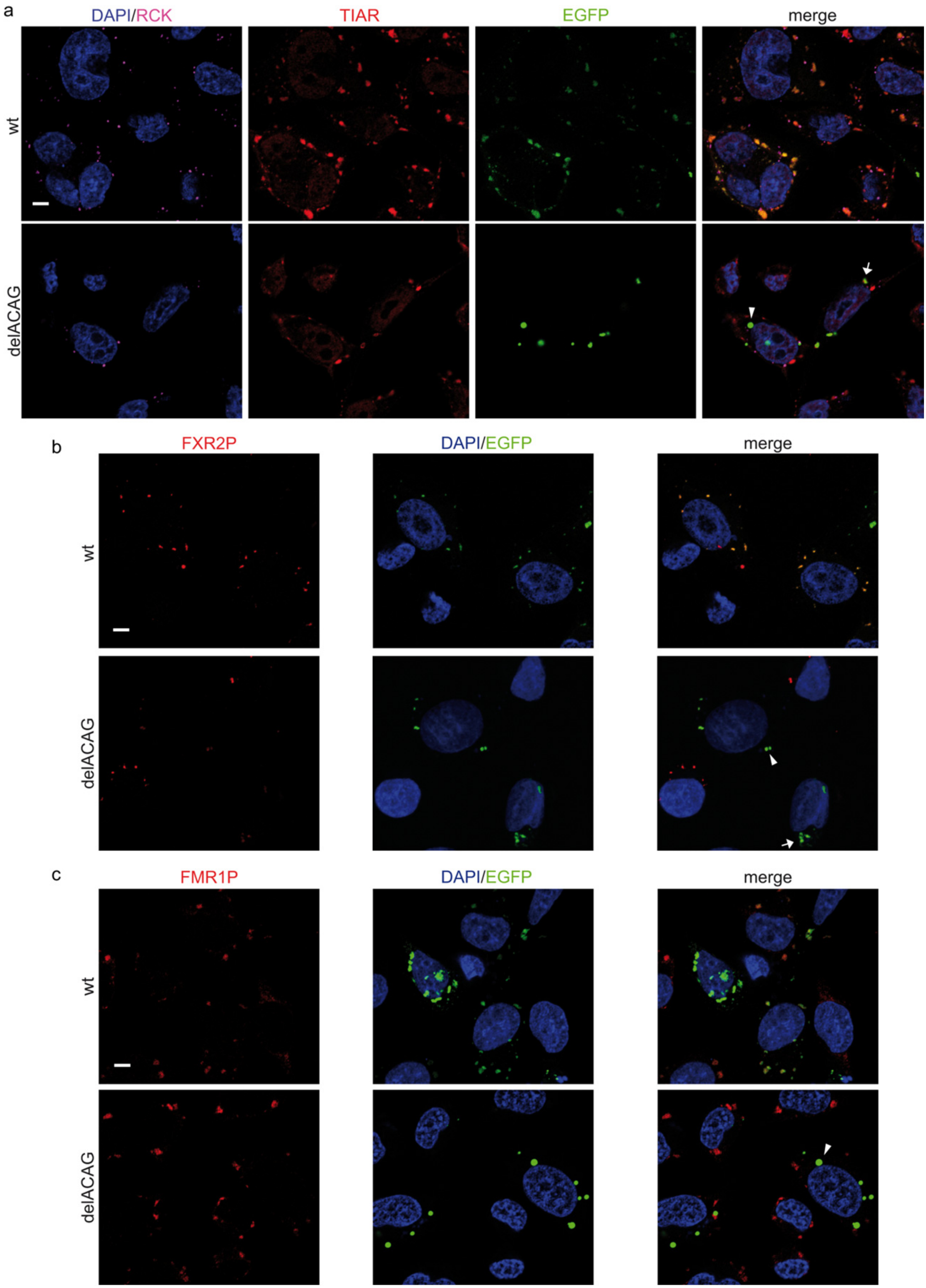

Supplementary Figure 11. Ring-shaped delACAG granules generated in transfected HeLa cells do not actively recruit SG components. a-c) Confocal immunofluorescence images (single Z-slice) of HeLa cells transfected with wt or delACAG EGFP:iso-e constructs, stressed with arsenite (ARS) and immunostained for the SG markers TIAR/RCK (a), FXR2P (b) or FMR1P (c). The EGFP signal from normal EGFP:iso-e-wt and mutant EGFP:iso-e-delACAG co-localizes with SG markers at morphologically irregular SGs (arrows), but there is no co-localization or weak co-localization (FXR2P, panel b) at delACAG ring-shaped granules (arrowheads). Equivalent results were obtained in HeLa cells transfected with EGFP:iso-e-delA or -dupA constructs. n=3. Scale bars 5 μm. Nuclei are stained with DAPI.

Supplementary Figure 12

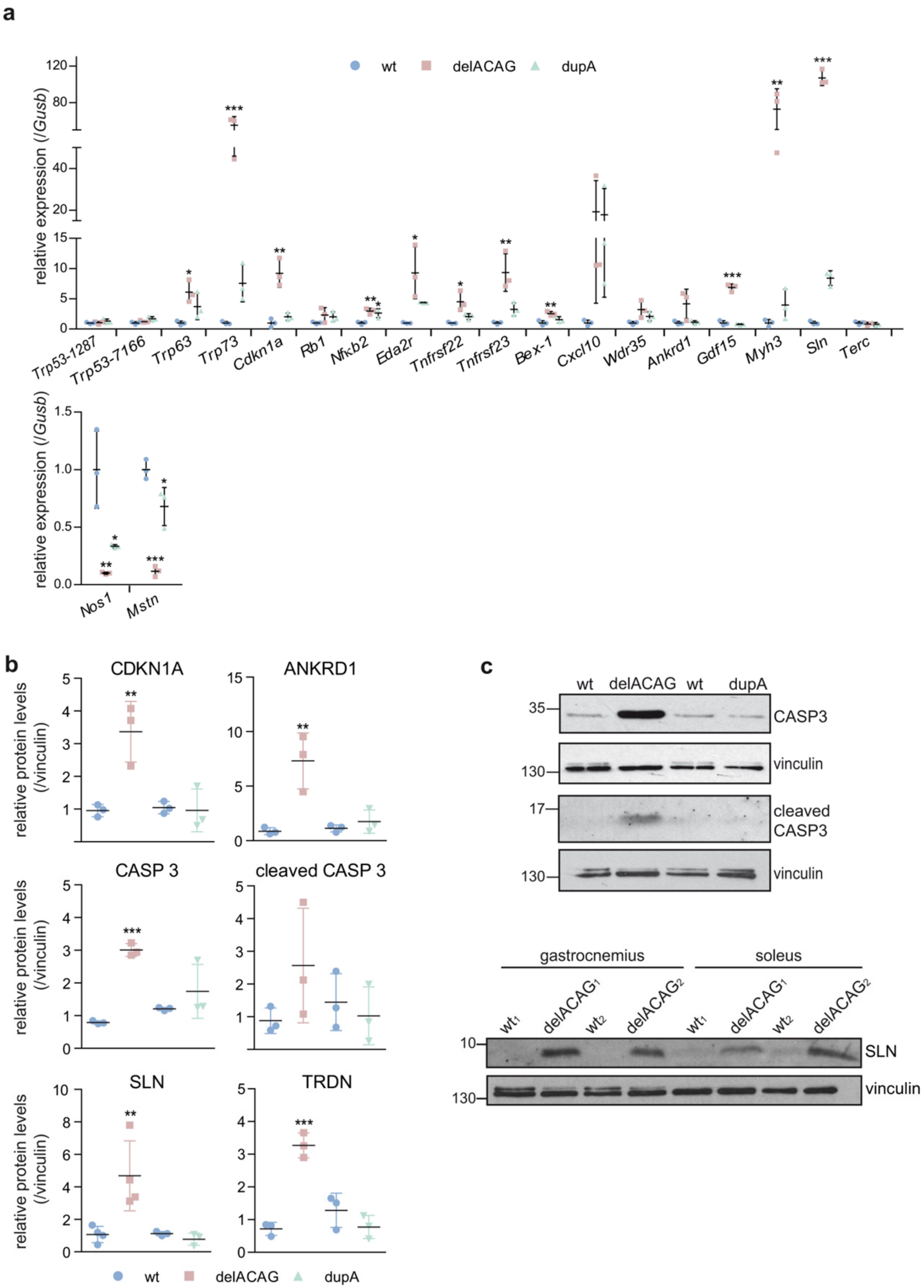

Supplementary Figure 12. **Gene expression analysis in skeletal muscle from exon-15 mutant mice.** a) Relative expression of selected DEGs by TaqMan qRT-PCR in gastrocnemius of wt, delACAG and dupA mice of 1.5 months of age using *Gusb* as the reference gene. Values are expressed as fold change of the mean value of wt mice. n=3. b) Quantification of CDKN1A, ANKRD1, CASP3, cleaved CASP3, SLN and TRDN protein levels in gastrocnemius (or soleus in the case of SLN) from delACAG, dupA mice and corresponding wt littermates determined by densitometry. Representative immunoblots are shown in Fig. 10 e-f. For each blot densitometric values were referred to vinculin and then normalized to the mean value of the total number of wt mice analysed within the same blot. n=7(wt), 4(delACAG), 3(dupA) for SLN and n=6(wt), 3(delACAG), 3(dupA) for the rest of proteins. Graphs in a-b represent mean ± s.d. followed by one-way ANOVA with Tukey post-hoc test. c) Top panel: replicate blots from experiments shown in Fig. 10 e-f using extracts from different mice. Lower panel: Additional immunoblot demonstrating protein levels of SLN in soleus and gastrocnemius of two different wt and delACAG mouse pairs at P21.Viculin is used as loading control.

Supplementary Figure 13

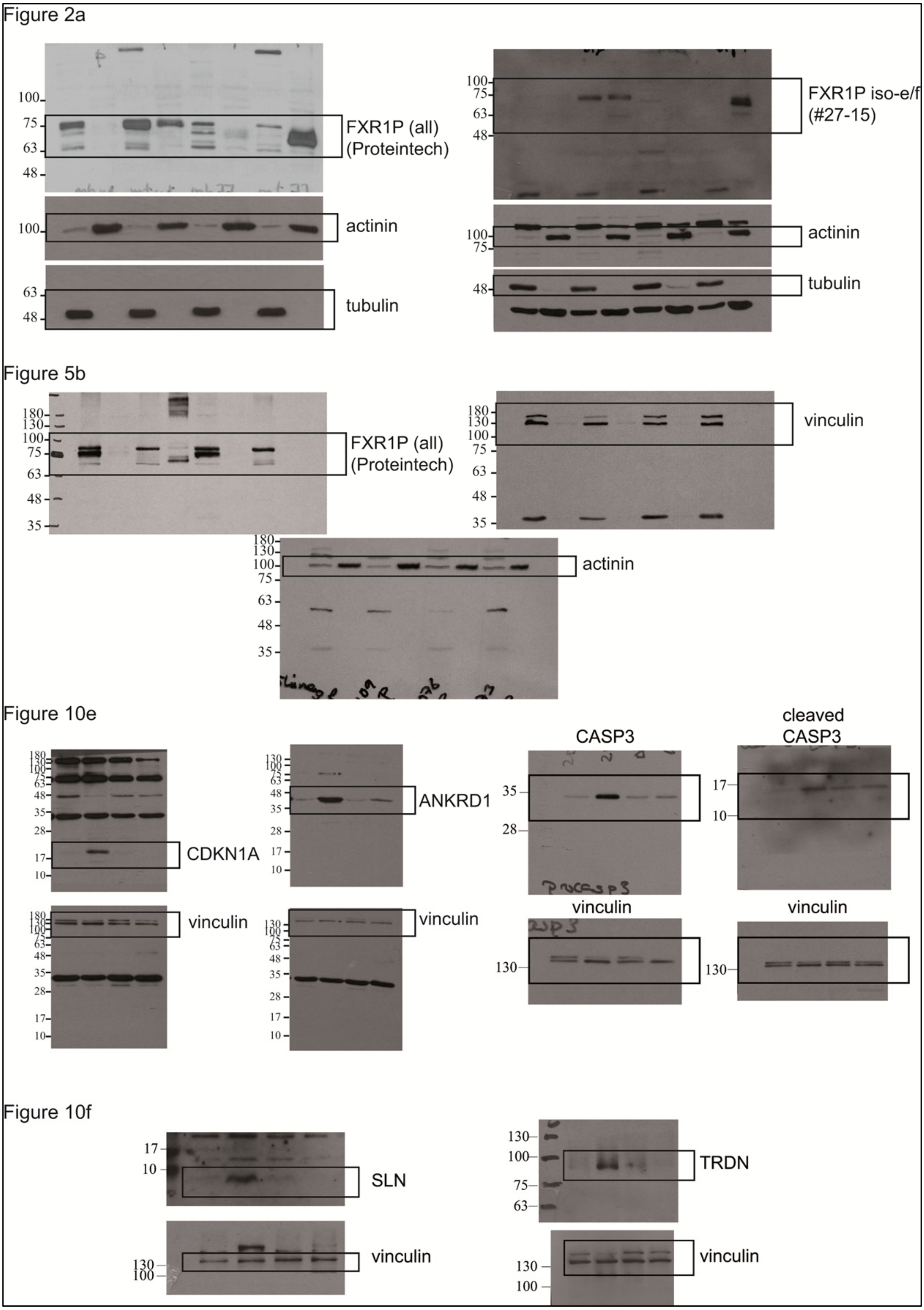

Supplementary Figure 3

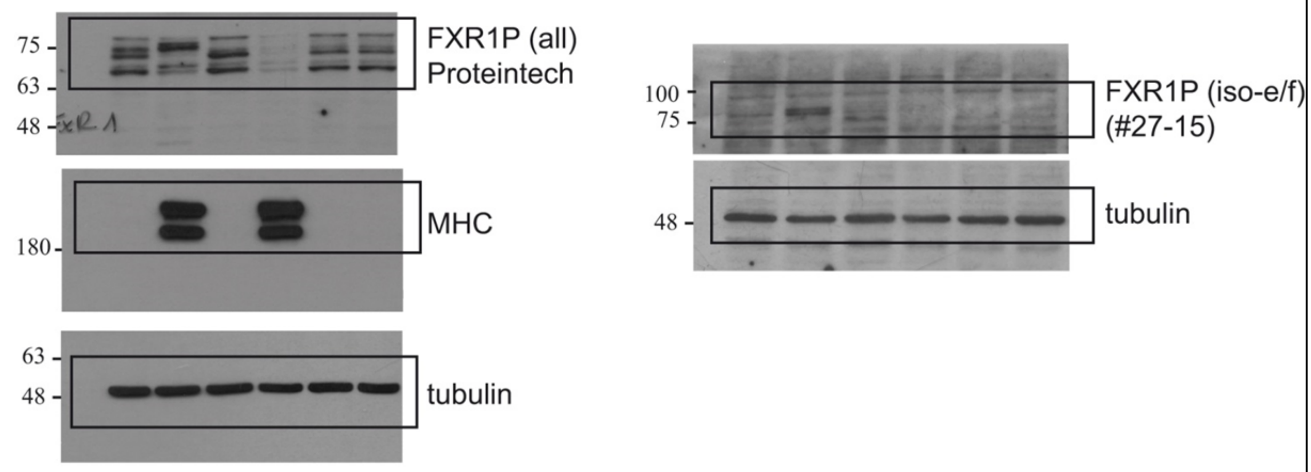

Supplementary Figure 4c

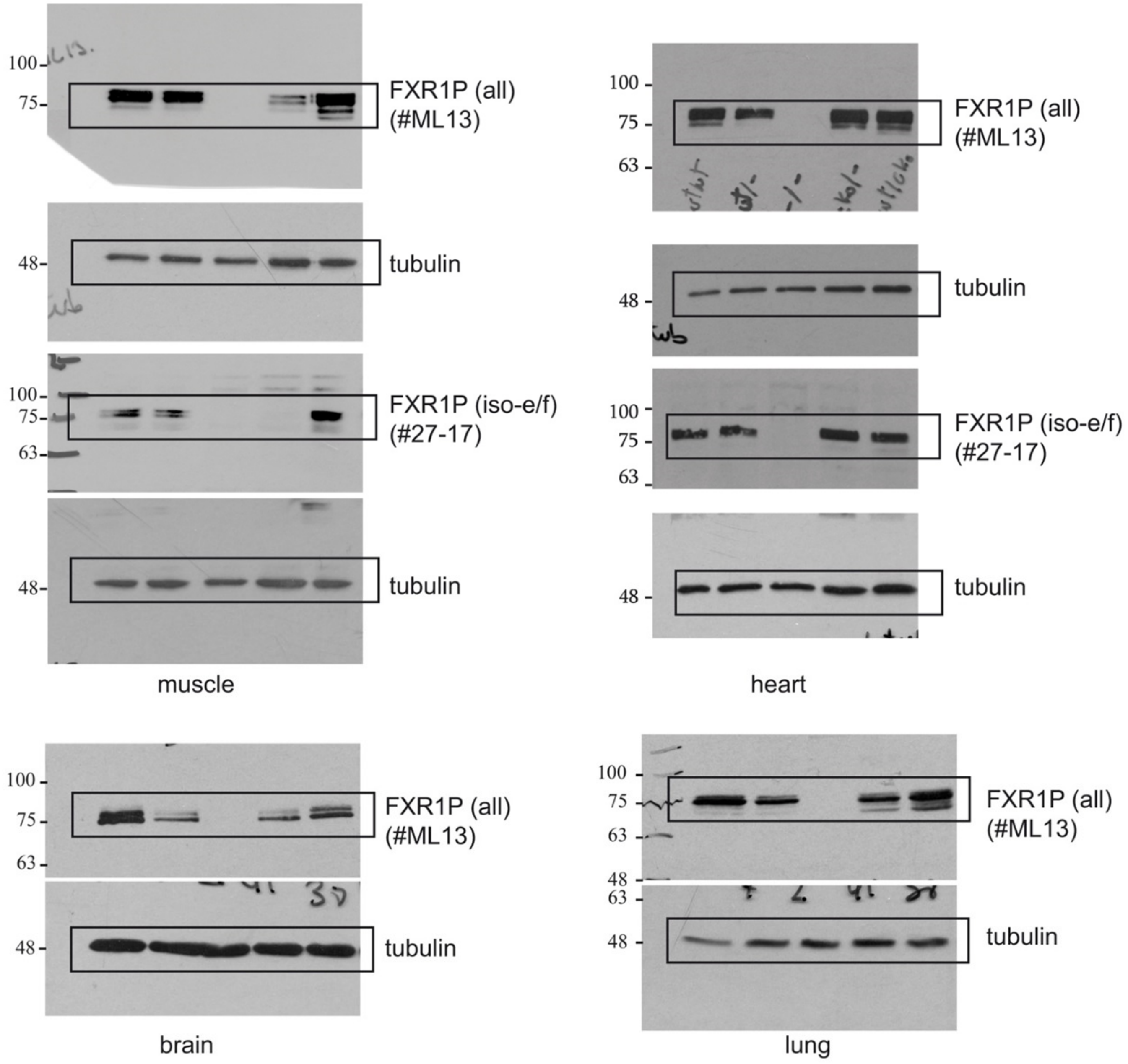

Supplementary Figure 7a

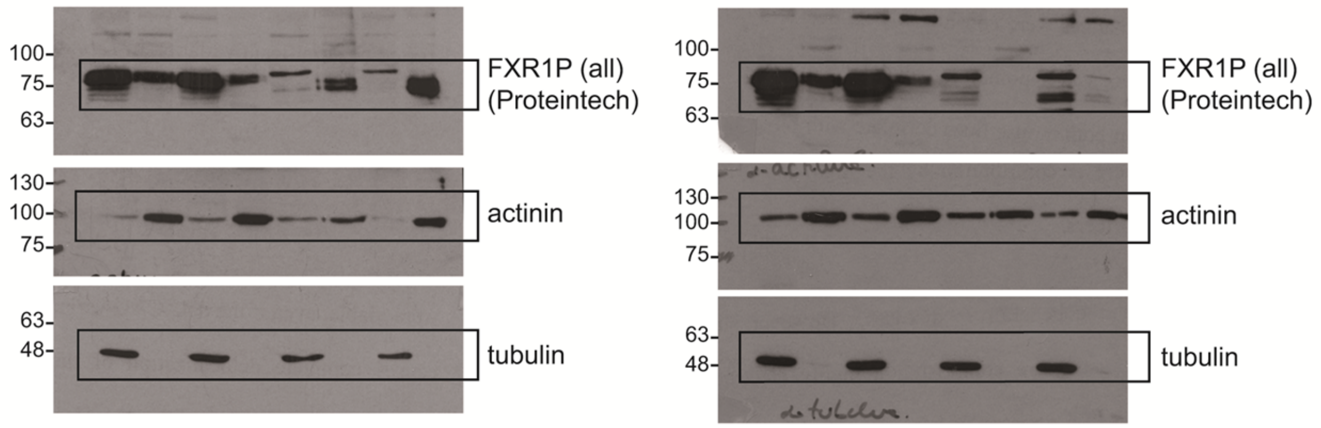

Supplementary Figure 10b

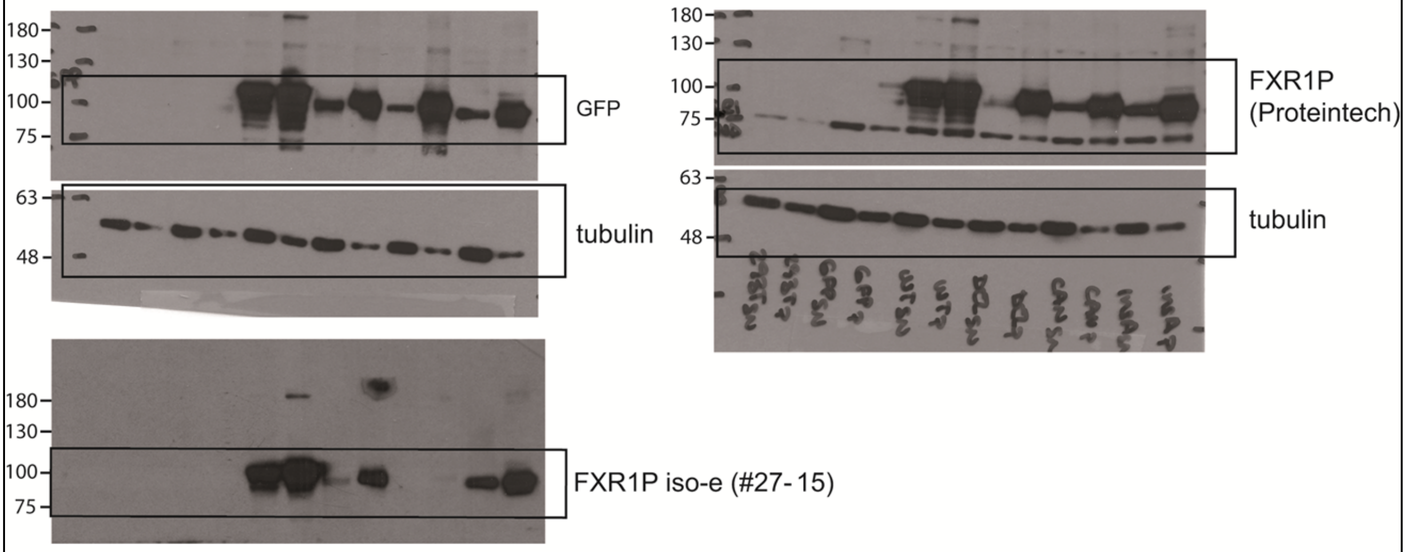

Supplementary Figure 12c

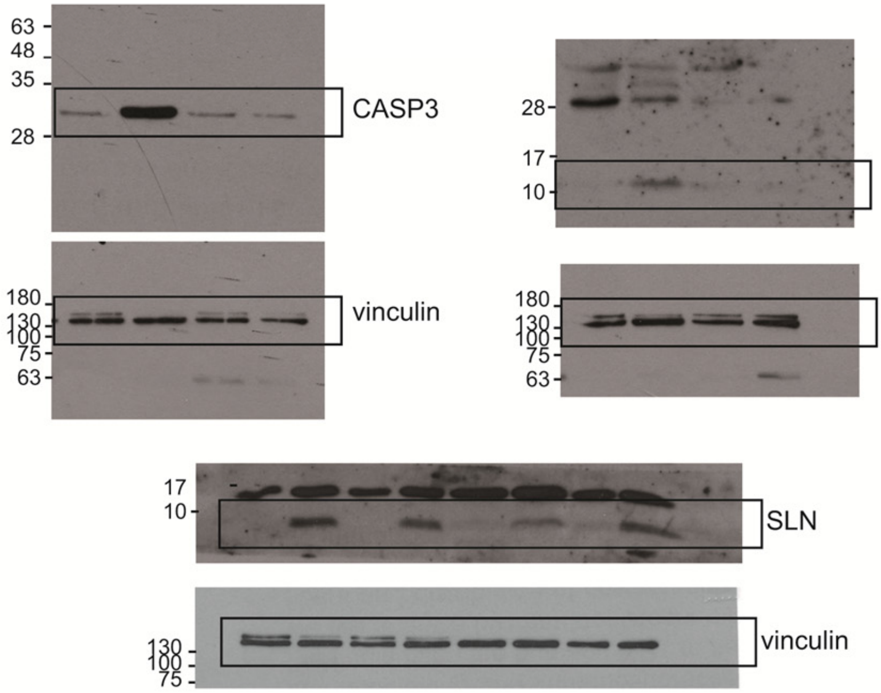

Supplementary Figure 13. Uncropped images of WBs. Uncropped scan of immunoblots inserted in different figures and supplementary figures of this manuscript. For each panel the number of the corresponding figure is indicated. Black boxes mark cropped specific bands shown in figures.

Supplementary Tables

Supplementary Table 1a. Pipeline used to analyse NGS-data of the proband of family 1

Filter	Number of variants
Numbers of variants called (coding and splice site variants, MAF≤ 0.05 1000 G)	5,031
Variant depth (Number of reads supporting the variant / Number of total reads) >=0.2	3,802
Variants with a MAF< 0.0001 (ENSEMBL release 75)	1,781
Neither in our in-house variant database nor ExAC with MAF > 0.0001	600
Number of variants inside a ROH	9

MAF: Minor Allele Frequency; ExAC: Exome Aggregation Consortium database; ROH: Regions of homozygosity.

Supplementary Table 1b. Candidate variants inside ROH identified in the proband of family 1

Chromosome	Position	Ref	Alt	Gene	Protein Effect	Ensembl protein ID	Polyphen Prediction	OMIM
3	180688933	TAGAC*	T	*FXR1*	R559Sfs*37	ENSP00000418097.1	N.P.	
11	123887188	G	C	*OR10G4*	D303H	ENSP00000325076.3	BENIGN	
15	40849476	G	A	*C15ORF57*	R114W	ENSP00000453429.1	PROB DAMAGING	
15	42744067	G	C	*ZNF106*	Q112E	ENSP00000263805.4	BENIGN	
15	44856848	T	C	*SPG11***	I2350V	ENSP00000261866.7	BENIGN	602099,616668,604360
20	30513704	G	A	*TTLL9*	R172Q	ENSP00000365086.2	POSS DAMAGING	
20	39980524	T	A	*LPIN3*	L389Q	ENSP00000362354.3	PROB DAMAGING	
22	51041662	C	G	*MAPK8IP2*	S61C	ENSP00000330572.3	BENIGN	
X	138827973	A	G	*ATP11C*	Y961H	ENSP00000332756.3	BENIGN	

Chromosome positions are referred to GRCh37/hg19 assembly. Reference (Ref) and alternative (Alt) alleles are indicated. Polyphen was used to predict the protein effect of each variant. N.P.: not predicted; PROB: Probably; POSS: Possibly. (*) Following the HGVS nomenclature guidelines the correct name of this variant at the cDNA level is: XM_005247813.3: c.1764_1767delACAG and XM_005247815.3: c.1677_1680delACAG for isoforms e and f respectively. (**) The homozygous missense change p.I2350V (gnomAD allele frequency: 2.438e-5) in the spastic paraplegia gene *SPG11*, classed as benign by Polyphen, was not present in V-4 after Sanger sequencing and thus was disregarded as the cause of the disease in family 1. Consistently, *SPG11* was not detected inside a ROH in V-4 (Supplementary Fig. 2a).

Databases of genetic variants (supplementary note 1 for acknowledgements):

dbSNP: http://www.ncbi.nlm.nih.gov/projects/SNP/

ExAC (Exome Aggregation Consortium)[4]: http://exac.broadinstitute.org/

ESP (Exome Variant Server, NHLBI GO Exome Sequencing Project (ESP), Seattle, WA): http://evs.gs.washington.edu/EVS/ [April, 2017 accessed]

1000 G (1000 Genomes Project)[5]: http://www.internationalgenome.org/home

gnomAD (Genome Aggregation Database)[4]: http://gnomad.broadinstitute.org/

Kaviar (~Known VARiants)[6] http://db.systemsbiology.net/kaviar/

Supplementary Table 2. Variants in multi-minicore genes (MAF<0.1) for family 1 and family 2

Chr	Position	Ref	Alt	Gene	Zygosity	Protein		Ref Seq	*rs#*	ExAC (MAF)	ClinVar
**FAMILY 1**											
**Recessive Minicore Genes**							**Shared by V-4 (parental inheritance)**				
2	179401845	A	G	*TTN*	Heterozygous	C33331R		ENSP00000467141.1	*rs56061641*	G=0.0003	Benign/Likely Benign/VUS
2	179418892	G	A	*TTN*	Heterozygous	S29649L	No (paternal)	ENSP00000467141.1	*rs764695663*	A=0.00003	VUS
2	179441707	G	A	*TTN*	Heterozygous	H23119Y	Yes, heterozygous (maternal)	ENSP00000467141.1	-	-	-
2	179477267	T	G	*TTN*	Heterozygous	N16662T		ENSP00000467141.1	*rs36043230*	G=0.0160	Benign/Likely benign
2	179578713	T	C	*TTN*	Heterozygous	N8891S		ENSP00000467141.1	*rs146057575*	C=0.0004	Benign/Likely Benign/VUS
12	121176113	A	T	*ACADS*	Heterozygous	T219S		ENSP00000242592.4	*rs144815059*	T=0.0002	-
19	38931451	G	A	*RYR1*	Heterozygous	A38T	No (paternal)	ENSP00000347667.3	*rs377558801*	A=0.00004	VUS
19	38976655	C	T	*RYR1*	Heterozygous	P1787L		ENSP00000347667.3	*rs34934920*	T=0.0196	Benign/Likely benign
19	38983180	G	T	*RYR1*	Heterozygous	G2060C		ENSP00000347667.3	*rs35364374*	T=0.0694	Benign
**Dominant Minicore Genes**											
16	772990	C	G	*CCDC78*	Heterozygous	W410S	Yes, heterozygous (maternal)	ENSP00000293889.6	*rs893572894* (multiallelic C/T/G)	T (W>Ter) =0.00003	-
**FAMILY 2**											
**Recessive Minicore Genes**							**Shared by all sibs (II-3, II-4, II-5)**				
19	38976655	C	T	*RYR1*	Heterozygous	P1787L	Yes, heterozygous	NM_000540.2	*rs34934920*	T=0.0196	Benign/Likely benign
2	179403750	C	T	*TTN*	Heterozygous	R31330H	No	NM_001256850.1	*rs4894028*	T=0.0680	Benign/Likely benign
2	179414162	T	C	*TTN*	Heterozygous	I29090V	No	NM_001256850.1	*rs16866391*	C=0.0313	-
2	179473201	G	A	*TTN*	Heterozygous	P15829L	No	NM_001256850.1	*rs372618781* (multiallelic A/G/T)	T(P>Q) =0.00003	(T) Benign/VUS
2	179497018	G	A	*TTN*	Heterozygous	R12894C	No	NM_001256850.1	*rs12471771* (multiallelic A/G/T)	A(R>C) =0.0210	Benign/Likely benign
2	179510739	A	G	*TTN*	Heterozygous	V11798A	No	NM_001256850.1	-	-	-
2	179589241	G	A	*TTN*	Heterozygous	A6637V	No	NM_001256850.1	*rs17355446*	A=0.0511	Benign/Likely benign

WES variants in previously reported recessive and dominant multi-minicore genes in the proband of family 1 and patients from family 2 (MAF<0.1). Variants classed as VUS (variant of unknown significance), or with no definition in ClinVar (NCBI), and particularly rare (MAF<0.01%) were analysed by Sanger sequencing in all members of family 1. One *TTN* variant (H23119Y) and the W410S variant in *CCDC78* were detected in the heterozygous state in both V-4 and the unaffected mother. The *CCDC78* variant involves a non-conservative amino acid, is multiallelic with one of the alleles resulting in W410Stop (MAF=0.00003), and relevantly, was also present in the unaffected mother. Moreover, this variant is classed as synonymous when a different reference sequence is used (*rs893572894*, dbSNP database (NCBI)). Chr: choromosome, Ref: reference allele, Alt: altered allele, Ref Seq: Reference sequence, rs#: variant identification number, ExAC (MAF): Minor Allele frequency in ExAC, ClinVar: Clinical Significance in ClinVar database (NCBI). For multiallelic variants MAF of one allele is indicated.

Supplementary Table 3a. Gene Ontology (GO) functional enrichment analysis of selected DEGs (differentially expressed genes)

ID	Description	GeneRatio	BgRatio	pvalue	p.adjust	geneID	Count
**GO:0072331**	signal transduction by p53 class mediator	10/217	125/20428	9E-07	0.002	Ankrd1/Bbc3/Cdkn1a/Eda2r/Mdm2/Phlda3/Smyd2/Trp63/Trp73/Zfp385a	10
**GO:0042770**	signal transduction in response to DNA damage	7/217	73/20428	1E-05	0.006	Ankrd1/Cdkn1a/Mdm2/Smyd2/Trp63/Trp73/Zfp385a	7
**GO:0044057**	regulation of system process	17/217	483/20428	2E-05	0.007	Adrb1/Camk2d/Cck/Cx3cl1/Dbh/Gas6/Inha/Kcnh2/Kcnn2/Mdm2/Mstn/Myl4/ Nos1/Rapgef4/Sln/Tlr4/Tmem100	17
**GO:0001836**	release of cytochrome c from mitochondria	6/217	57/20428	3E-05	0.009	Bbc3/Cck/Mmp9/Sfn/Trp73/Wdr35	6
**GO:0097193**	intrinsic apoptotic signaling pathway	12/217	274/20428	4E-05	0.01	Bbc3/Casp3/Cdkn1a/Eda2r/Mdm2/Mmp9/Phlda3/Scn2a/Sfn/Trp63/Trp73/Zfp385a	12
**GO:2001233**	regulation of apoptotic signaling pathway	14/217	382/20428	6E-05	0.012	Atf3/Bbc3/Cx3cl1/Dbh/Gdnf/Mdm2/Mmp9/Tnfrsf22/Tnfrsf23/Trp63/Trp73/Wdr35/ Wnt4/Zfp385a	14
**GO:0044819**	mitotic G1/S transition checkpoint	4/217	21/20428	6E-05	0.012	Cdkn1a/Mdm2/Trp63/Trp73	4
**GO:0071214**	cellular response to abiotic stimulus	10/217	211/20428	9E-05	0.012	Ankrd1/Bbc3/Cdkn1a/Cers1/Mdm2/Nos1/Scn2a/Trp63/Trp73/Wnt11	10
**GO:0008015**	blood circulation	15/217	444/20428	9E-05	0.013	Acta2/Actc1/Adrb1/Alox12/Camk2d/Cx3cl1/Dbh/Gas6/Kcnh2/Kcnn2/Mdm2/Myl4/ Nav2/Nos1/Wdr35	15
**GO:0048485**	sympathetic nervous system development	4/217	23/20428	9E-05	0.013	Gdnf/Plxna4/Sox11/Trp63	4
**GO:0001508**	action potential	8/217	136/20428	1E-04	0.013	Camk2d/Gria1/Kcnb1/Kcnh2/Kcnn2/Rapgef4/Scn2a/Scn3a	8
**GO:0052646**	alditol phosphate metabolic process	3/217	10/20428	1E-04	0.015	Gpd1/Gpd2/Slc37a2	3
**GO:0048483**	autonomic nervous system development	5/217	47/20428	1E-04	0.015	Gdnf/Nav2/Plxna4/Sox11/Trp63	5
**GO:0043491**	protein kinase B signaling	8/217	156/20428	3E-04	0.024	AI464131/Cd28/Gas6/Mstn/Phlda3/Rras/Sesn3/Tmem100	8
**GO:0030858**	positive regulation of epithelial cell differentiation	5/217	57/20428	3E-04	0.028	Alox12/Foxj1/Gdnf/Sfn/Tmem100	5
**GO:0060537**	muscle tissue development	13/217	408/20428	4E-04	0.033	Actc1/Adrb1/AI464131/Ankrd1/Atf3/Camk2d/Kcnk2/Klf5/Mstn/Rb1/Sox11/ Trp63/Trp73	13
**GO:0051899**	membrane depolarization	6/217	92/20428	4E-04	0.033	Alox12/Camk2d/Cck/Kcnh2/Scn2a/Scn3a	6
**GO:0007599**	hemostasis	8/217	176/20428	6E-04	0.04	Alox12/Anxa8/Cx3cl1/Gas6/Myl9/Rab27a/Tlr4/Zfp385a	8
**GO:0034764**	positive regulation of transmembrane transport	7/217	135/20428	6E-04	0.04	Adrb1/Car2/Cxcl10/Kcnh2/Nos1/Slc34a1/Wnk2	7
**GO:0006936**	muscle contraction	10/217	271/20428	7E-04	0.042	Acta2/Actc1/Adrb1/Camk2d/Gdnf/Kcnh2/Kcnn2/Myh3/Myl4/Nos1	10
**GO:0046827**	positive regulation of protein export from nucleus	3/217	17/20428	7E-04	0.043	Gas6/Mdm2/Sfn	3
**GO:0032715**	negative regulation of interleukin-6 production	4/217	43/20428	0.001	0.058	Foxj1/Gas6/Nlrc3/Tlr4	4
**GO:0042692**	muscle cell differentiation	12/217	399/20428	0.001	0.058	Actc1/Adrb1/AI464131/Ankrd1/Camk2d/Cxcl10/Gdf15/Krt8/Lmod2/Mstn/Rb1 /Wnt4	12
**GO:0019228**	neuronal action potential	4/217	45/20428	0.001	0.058	Gria1/Rapgef4/Scn2a/Scn3a	4
**GO:0048486**	parasympathetic nervous system development	3/217	21/20428	0.001	0.058	Gdnf/Nav2/Plxna4	3
**GO:0003015**	heart process	8/217	200/20428	0.001	0.058	Actc1/Adrb1/Camk2d/Kcnh2/Kcnn2/Mdm2/Myl4/Nos1	8
**GO:0035914**	skeletal muscle cell differentiation	5/217	73/20428	0.001	0.058	Ankrd1/Atf3/Klf5/Rb1/Sox11	5
**GO:0070252**	actin-mediated cell contraction	5/217	77/20428	0.001	0.058	Actc1/Adrb1/Camk2d/Kcnh2/Kcnn2	5

GO terms ordered by pvalue. Only GO terms with count ≥4 are represented in Fig. 10c.

Supplementary Table 3b. KEGG enrichment analysis of selected DEGs

ID	Description	GeneRatio	BgRatio	pvalue	p.adjust	qvalue	geneID	Count
**mmu04115**	p53 signaling pathway	7/89	67/7758	1,02539E-05	0,001999505	0,001716175	Bbc3/Casp3/Cdkn1a/Mdm2/Sesn3/Sfn/Trp73	7
**mmu05205**	Proteoglycans in cancer	10/89	201/7758	9,32578E-05	0,009092634	0,007804204	Camk2d/Casp3/Cdkn1a/Fzd10/Mdm2/Mmp9/ Rras/Tlr4/Wnt11/Wnt4	10

Supplementary note 1. Acknowledgements

The authors would like to thank:

The Exome Aggregation Consortium and the groups that provided exome variant data for comparison. A full list of contributing groups can be found at http://exac.broadinstitute.org/about.

The NHLBI GO Exome Sequencing Project and its ongoing studies which produced and provided exome variant calls for comparison: the Lung GO Sequencing Project (HL-102923), the WHI Sequencing Project (HL-102924), the Broad GO Sequencing Project (HL-102925), the Seattle GO Sequencing Project (HL-102926) and the Heart GO Sequencing Project (HL-103010).

The 1000 Genomes Project and the groups that provided exome variant data for comparison.

The Genome Aggregation Database (gnomAD) and the groups that provided exome and genome variant data to this resource. A full list of contributing groups can be found at http://gnomad.broadinstitute.org/about.

The creators of Kaviar for providing a useful tool for comparing variant data.

Suplementary References

1. Dube, M., Huot, M.E. & Khandjian, E.W. Muscle specific fragile X related protein 1 isoforms are sequestered in the nucleus of undifferentiated myoblast. *BMC Genet.* **1**, 4 (2000).

2. Kirkpatrick, L.L., McIlwain, K.A. & Nelson, D.L. Alternative splicing in the murine and human FXR1 genes. *Genomics* **59**, 193-202 (1999).

3. Nagy, E. & Maquat, L.E. A rule for termination-codon position within intron-containing genes: when nonsense affects RNA abundance. *Trends Biochem. Sci.* **23**, 198-199 (1998).

4. Lek, M., *et al.* Analysis of protein-coding genetic variation in 60,706 humans. *Nature* **536**, 285-291 (2016).

5. Genomes Project, C., *et al.* A global reference for human genetic variation. *Nature* **526**, 68-74 (2015).

6. Glusman, G., Caballero, J., Mauldin, D.E., Hood, L. & Roach, J.C. Kaviar: an accessible system for testing SNV novelty. *Bioinformatics* **27**, 3216-3217 (2011).
