# Supplementary material for: Recessive mutations in muscle-specific isoforms of FXR1 cause congenital multi-minicore myopathy
Source: Nat Commun. 2019 Feb 15;10:797. doi: 10.1038/s41467-019-08548-9 (PMC6377633; doi:10.1038/s41467-019-08548-9)
Supplement: Supplementary file 4 — Description of Additional Supplementary Files [file 41467_2019_8548_MOESM4_ESM.docx]

**Title:** Supplementary Data 1
**Description:** Detailed clinical description of patients
